# Caregiver Needs: Navigating Services, Technology, and Information

**DOI:** 10.1093/geroni/igaa057.2375

**Published:** 2020-12-16

**Authors:** C Grace Whiting

**Affiliations:** National Alliance for Caregiving, Washington, District of Columbia, United States

## Abstract

Data suggests that caregivers may be taking on this role without adequate and affordable services and supports in place. Few caregivers report access to paid help and find it difficult to navigate a healthcare system that is complex and that changes continuously. Caregivers find it challenging to coordinate their recipients’ care across various providers, and the lack of affordable services make it difficult to be a caregiver today. While many caregivers rely on health care professionals (such as doctors, nurses, or social workers) as a source of information about providing care, few caregivers report having conversations with them about what they need to care for their recipient or to support their well-being. In this section, the presenter will discuss the needs of caregivers (respite, transportation, information, and training), focusing on policy and programmatic solutions geared to improving the well-being of caregivers and those under their care.

